# Assessing of the contributions of pod photosynthesis to carbon acquisition of seed in alfalfa (*Medicago sativa* L.)

**DOI:** 10.1038/srep42026

**Published:** 2017-02-07

**Authors:** Wenxu Zhang, Peisheng Mao, Yuan Li, Mingya Wang, Fangshan Xia, Hui Wang

**Affiliations:** 1 Key Laboratory of Eco-Environment-Related Polymer Materials, Ministry of Education of China, Key Laboratory of Gansu Polymer Materials, College of Chemistry and Chemical Engineering, Northwest Normal University, Lanzhou 730070, China; 2 Institute of Grassland Science, China Agricultural University, Beijing 100193, China; 3 Dryland Farming Institute, Hebei Academy of Agricultural and Forestry Sciences, Hengshui 053000, China

## Abstract

The distribution of carbon from a branch setting pod in alfalfa was investigated during the seed development of seeds to determine the relative contribution of pod and leaf photoassimilates to the total C balance and to investigate the partitioning of these photoassimilates to other plant organs. A ^13^Clabeling procedure was used to label C photoassimilates of pods and leaves in alfalfa, and the Δ^13^C values of a pod, leaves, a section of stem and roots were measured during seed development on day 10, 15, 20 and 25 after labeling of the pod. The results showed that the alfalfa pod had photosynthetic capacity early in the development of seeds, and that pod photosynthesis could provide carbon to alfalfa organs including seeds, pods, leaves, stems and roots, in addition to leaf photosynthesis. Photosynthesis in the pod affected the total C balance of the alfalfa branch with the redistribution of a portion of pod assimilates to other plant organs. The assimilated ^13^C of the pod was used for the growth requirements of plant seeds and pods. The requirements for assimilated C came primarily from the young pod in early seed development, with later requirements provided primarily from the leaf.

Alfalfa (*Medicago sativa* L.) is the most widely cultivated forage legume in temperate areas and is a predominant component in mixtures for hay, silage and pasture. In China, more than 1.6 million hm^2^ are planted to alfalfa, with the agronomic value primarily in cultivation for forage production rather than seed yield. However, the ability of a cultivar to provide a high seed yield determines the competitive selling price, which is a key factor for the effective distribution of a cultivar to farmers^1^. The demand for alfalfa seed has gradually increased and is expected to continue, reaching 30,000–40,000 t in the next 5 years in China. However, at current levels, the domestic seed production would only meet approximately one-third of the future demand. Because of the characters of perennial growth and indefinite inflorescence, the seed yield of alfalfa is low, which limits the distribution of cultivars; consequently, the alfalfa seed yield is low and unstable (201–915 kg ha^−1^) in China^2^. Therefore, to improve the yield of alfalfa seeds, research on the development and maturity of seeds is urgently required.

Most of the crop yield, 90–95%, is the result of photosynthesis. Although green leaves are generally the primary source of photosynthetic production, reproductive organs that are photosynthetically active can also determine yields^3^,^4^. Some non-leaf organs contain chlorophyll and have photosynthetic capacity, including the pod in rapeseed (*Brassica napus* L.), garden pea (*Pisum sativum*) and soybean (*Glycine max* L.)^5^,^6^,^7^,^8^,^9^,^10^,^11^. As primarily heterotrophic organs, seeds are dependent on nutrients supplied by the photosynthetic organs for the synthesis of reserves during development. In studies of pea, a significant portion of seed carbon is assimilatedby the pod^7^,^12^. Similar to the pea, does the pod in alfalfa have photosynthetic capacity, and does pod photosynthesis contribute to seed production in alfalfa? These questions remain unanswered because pod photosynthesis in alfalfa has not been investigated.

Stable C isotope tracers are a key tool to study C management in plants and the implications of photosynthetic performance^13^. In some studies, labeling with ^13^C/^12^C is used in the characterization of assimilated C and the further partitioning into different organs^14^,^15^.

This experiment was designed to investigate the changes in ^13^CO_2_ assimilation in both pods and leaves during the stages of seed development in alfalfa, and the following questions were addressed: (i) Did the alfalfa pod have photosynthetic capacity? (ii) What were the contributions during the growth of the pod and leaves to the total C balance? and (iii) How was the partitioning of ^13^C assimilated by the pod and leaves affected by the growth requirements of seeds.

## Results

### Changes in Δ^13^C values among plant organs

**Figure 1:**
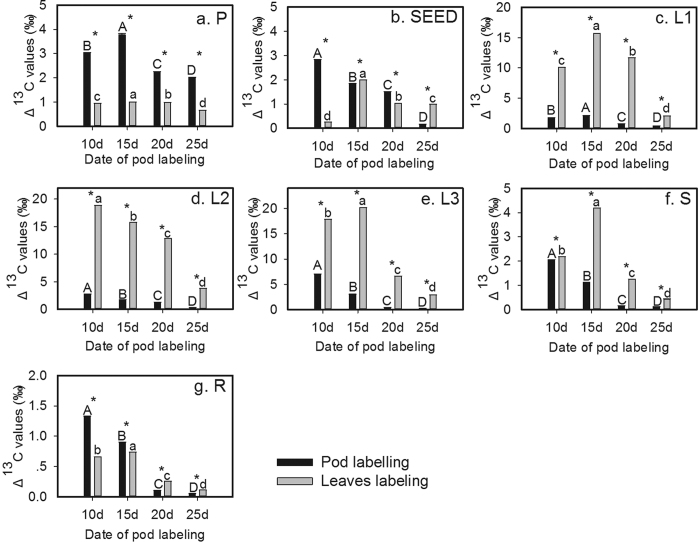
Changes for Δ^13^C values (‰) in the alfalfa organs (P, SEED, L1, L2, L3, S and R) according to the development stage (from day10 to 20 after podding) after pod or leaves labeling. Each value represents the mean ± SE (n = 3). The different letters indicate significant differences (*P* < 0.01) among treatments as determined by the Tukey b-test. The capital and lowcase letter indicates pod and leaves labeling, respectively. *Indicate significant differences (*P* < 0.01) between pod and leaves labeling of samples as determined by the T test.

A result of ^13^C labeling of the pod or leaves, the Δ^13^C values were significantly different (*P* < 0.01) in the different alfalfa organs P (pod), SEED(seeds), LI (the first leaf under the pod), L2 (the second leaf under the pod), L3 (the third leaf under the pod), S (the first internode length of stem under the third leaf) and R (10 cm of roots) at each stage of seed development (Fig. 1). Obviously, the Δ^13^C values of pods at each stage of development were higher when the pod was labeled compared with the labeling of leaves (Fig. 1a), with similar higher values also appearing in the seeds from the labeled pod at the10 and 20 d stages after podding (Fig. 1b). However, the Δ^13^C values at each stage in the leaves and stem were lower when the pod was labeled than when leaves were labeled (Fig. 1c,d,e and f). Values were also lower in roots from the labeled pod from day 20 to 25 after podding (Fig. 1g) (Figure S1).

Following the labeling of the pod, significant changes (*P* < 0.01) were detected in samples from day 10 to 25 after podding in alfalfa (Fig. 1). The Δ^13^C values of samples decreased with time following pod labeling, except for the P and L1 in which the highest values were reached 15 d after podding, before gradually decreasing. Therefore, the highest assimilation of ^13^C in the samples occurred at the 10 d stage, except for P and L1 with the highest assimilation at the 15 d stage. At 25 d after podding, the lowest assimilation of ^13^C was detected in the samples.

For the samples labeled by leaves, Δ^13^C values changed significantly (*P* < 0.01) from day10 to 25after podding in alfalfa, with values first increasing and then decreasing, except in the L2 (Fig. 1). Furthermore, the highest values of ^13^C assimilated in the samples occurred at the15 d stage, except for the L2, which had the highest value at the 10 d stage. At 25 d after podding, the lowest assimilation of ^13^C was observed in the samples.

### Contribution of the pod and leaves to the carbon in each organ

From the labeled pod and leaves, each organ accumulated new C from day 10 to 25 after podding; however, the source of the accumulation of new C in each organ varied with the development of seed. The contribution rate (CR) is the portion of new C accumulated in the organs provided by photosynthesis in the pod or leaves (Figure S2).

**Figure 2:**
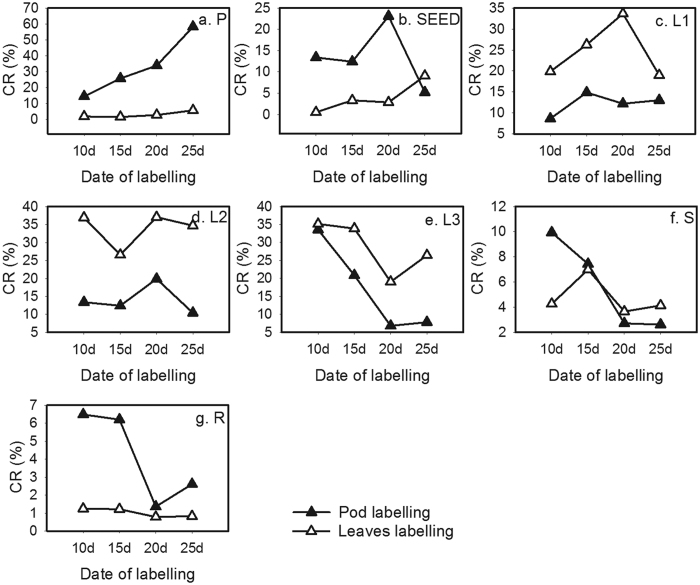
Changes for the CR of alfalfa organs (P, SEED, L1, L2, L3, S and R) according to the development stage (from day10 to 20 after podding) after pod or leaves labeling.

In the P organ, the CR increased gradually after labeling of the pod from day10 to 25 after podding. However, when leaves were labeled, the CR first decreased and then increased, and the rangeability was slower than that after pod labeling (Fig. 2a). At each stage, the CR of the labeled pod was higher than that of labeled leaves. From day10 to 25 after podding, the CR of pod labeling increased to 43.71%, whereas that of leaf labeling was 3.84%. The mean CR of the labeled pod and leaves was 33.14% and 3.97%, respectively.

In the SEED organ, the CR of the labeled pod showed a pattern of undulation, with first a slow decrease from stage 10 d to stage 15 d after podding and then a rapid increase from stage 15 d to stage 20 d, followed by another rapid decrease after day 20. However, the CR of the labeled leaves showed the opposite pattern. At each stage, the CR of the labeled pod was higher than that of labeled leaves, except at 25 d after podding (Fig. 2b). The CR of pod labeling reached the highest value (23.08%) at day 20 after podding, whereas the highest value of leaf labeling was 9.09% at 25 d after podding. The mean CR of the labeled pod (13.52%) was higher than the CR of labeled leaves (3.96%).

In the L1 organ, the CR of the labeled pod fluctuated and first increased from stage 10 d to stage 15 d after podding, decreased from stage 15 d to stage 20 d and then increased again after stage 20 d. However, the CR of labeled leaves first increased from stage 10 d to stage 20 d after podding and then decreased sharply. The change in the CR of the labeled pod was slower than that when leaves were labeled. At each stage, the CR value of pod labeling was lower than that of leaf labeling (Fig. 2c). The CR of the labeled pod reached the highest value (14.88%) at day15 after podding, where as the highest CR of the labeled leaves was 33.68% at day 20 after podding. The mean CR of the labeled pod (12.20%) was less than half the CR of labeled leaves (24.72%).

In the L2 organ, the pattern of change in the CR was similar for the labeled pod and leaves during the development from day10 to 25 after podding. First, a decrease was observed from stage 10 d to stage 15 d after podding, which was followed by an increase at day20, before decreasing again. At each stage, the CR of the labeled pod was lower than that of labeled leaves (Fig. 2d). The highest CR value for both the labeled pod and leaves (19.91% and 10.43%, respectively) was at stage 20 d after podding. The mean CR of labeled leaves (33.83%) was 2.4-fold higher than that of labeled pods (14.04%).

In the L3 organ, the pattern of change was similar for the CR of pod and leaf labeling during development from stage 10 d to stage 25 d after podding; the CR first decreased from day10 to 20 and then increased. However, the CR value of the labeled pod decreased sharply, and at each stage, the CR of labeled leaves was higher than that of the labeled pod (Fig. 2e). The CR of the labeled pod decreased from the highest (33.57%) to the lowest (6.79%) value, whereas the CR of labeled leaves changed from 35.17% at stage 10 d to 19.06% at stage 20 d after podding. The mean CR of the labeled pod (17.26%) was approximately 60% of the CR of labeled leaves (28.64%).

In the S organ, the CR of the labeled pod decreased rapidly from stage 10 d to stage 20 d after podding. However, the CR pattern of labeled leaves undulated and first increased at stage 15 d after podding and then decreased at stage 20 d before increasing again. The CR of the labeled pod was higher than that of labeled leaves at stages10 d and 15 d after podding but was lower than that of labeled leaves at stages 20 d and 25 d (Fig. 2f). The CR of the labeled pod decreased 7.33% from day 10 to 25 after podding. The mean CR of labeled leaves (4.76%) was lower than that of the labeled pod (5.68%).

In the R organ, the pattern of change was similar for the CR of the labeled pod and leaves during development from stage 10 d to stage 25 d after podding. The CR first decreased from day10 to 20 after podding and then increased. At each stage, the CR of the labeled pod was higher than that of labeled leaves (Fig. 2g). The CR of the labeled pod decreased by 5.12% and that of labeled leaves by 0.46% from day 10 to 20 after podding. The mean CR of labeled leaves (1.02%) was approximately 25% of the labeled pod CR (4.16%).

## Discussion

Alfalfa is a perennial forage legume that grows several years with root regrowth in the spring. However, the level of seed yield is limited by the characteristics of perennial growth, and the yield of seeds from a perennial crop is usually much lower than that of an annual crop. For seed development, it is important that sufficient assimilate is transferred to the seeds. In this study, based on the Δ^13^C values from the labeling of pods and leaves, the requirements for C in the organs, including the pod, seeds, leaves, stem and roots, were all concentrated within 15 d after podding. The assimilated ^13^C in the pod was primarily sourced from the labeled pod (mean 2.77‰), with little contribution from the labeled leaves (mean 0.90‰), whereas the assimilated ^13^C in the leaves (L1, L2 and L3) and stem was primarily from the labeled leaves. In the leaves, the mean Δ^13^C value from labeled leaves was 4–8-fold higher than that from the labeled pod. However, for the source of ^13^C in the seeds, a complete change occurred during development, and the ^13^C value from the labeled pod decreased significantly (*P* < 0.01) and that from labeled leaves increased significantly (*P* < 0.01) at 15 d after podding. Therefore, the C required for seed development within 10 d after podding was primarily from the pod, whereas that required within 15 to 25 d after podding was primarily from the leaf. The mean Δ^13^C values from the labeled pod and leaves in seeds showed that the ^13^C assimilated from the pod was determined by the growth requirements of seeds within the plant. Thus, photosynthesis in both the pods and leaves met the C requirements of the seeds in alfalfa. The pod significantly affected the C balance of alfalfa by redistributing most of its assimilates to the other organs (P, SEED, L1, L2, L3, S and R). Photosynthesis is documented in other reproductive organs (such as greenish flowers or developing fruits) in related research. Vaillant-Gaveau *et al*.^16^ found that 29% of C assimilated by the inflorescence in *Vitis vinifera* L. remained in the inflorescence, whereas the portion partitioned to the stem reached 42%, with lower proportions of 15% in leaves and 14% in roots^16^. The photosynthetic contribution of the pod to seed yield is approximately 70–100% in *Brassica*^9^,^10^. The Δ^13^C values of SEED were significantly different from stage10 d to stage 25 d after podding (*P* < 0.01), most likely because storage and accumulation in seeds is determined by intrinsic traits of the seed (sink strength) and the availability of assimilates to the developing seed (source strength)^17^.

For the CR based on the Δ^13^C values in the different leaves of alfalfa (L1, L2 and L3), the changes were similar, and the Δ^13^C and CR values from labeled leaves were all higher than those from the labeled pod, particularly within 15 d after podding. As this result showed, the photosynthetic capacity of the leaf was higher than that of the pod, and the leaf was the primary photosynthetic organ in alfalfa. However, the CR from labeled leaves in the pod and seed was lower than that from the labeled pod, except for the CR in the seed at 25 d after podding. Furthermore, the CR of the labeled pod in the pods reached the maximum (58.26%) at 25 d after podding, and the mean CR of the labeled pod (33.14%) was 8.3-fold higher than that of labeled leaves (3.97%) in the pod during seed development. A similar result is found in *Brassica campestris* and *B. napus*^10^,^18^. However, because *Brassica* leaves drop as the seeds develop, the photosynthetic contribution of the pod is higher than that of alfalfa. Additionally, in the present study, the newly assimilated C in the pod primarily came from self-fixed C, and the newly assimilated C in the seeds also primarily came from the young pod during early development. However, in the later stage of seed development, photosynthesis in the leaves provided the C. A similar phenomenon is observed with *Brassica* in which the photosynthetic rate of the pod has a triphasic pattern. In the first phase, which continues up to 20 days after anthesis, the rate of photosynthesis of the pod increases; in the second phase (20–40 days after anthesis), the rate remains more or less constant; andin the last phase (40–50 days after anthesis), the rate decreases^9^. Additionally, at the full-bloom stage, leaves near the flowers start to grow and with the rapid increase in the functional leaf area, sufficient assimilate is transported to the seeds and other organs. In the present study, the C assimilated by the pod that was translocated to organs decreased with time, particularly from the pod to the seeds. However, this decrease in translocation from the pod is a natural phenomenon, because with the beginning of pod senescence and initiation of maturation, the functional pod area declines rapidly. The pod was both a strong C source that translocated most of the newly assimilated C to the seed and a strong sink for C that accumulated less of the newly assimilated C to ensure its growth. Thus, photosynthesis in the pod was associated with the stages of growth, nutrient and matter accumulation and maturation in the development of seeds^19^,^20^.

In this study, the total Δ^13^C values of pods and leaves were 50.27‰ and 159.63‰, respectively; however, the mean CR of the pod (13.52%) was approximately 3.4-fold higher than that of leaves (3.96%) for all stages. In alfalfa, more ^13^C assimilated by the pod was distributed to seeds than ^13^C assimilated by leaves. Thus, the growth requirements of seeds were provided by pod photosynthesis. For the contributions of C, a high proportion of newly assimilated C is transported to sinks such as the pod, seeds and leaves^21^. However, in alfalfa, the CR was clearly different for the two sources of C: the CR of the labeled pod in the root was higher than that of labeled leaves within 15 d after podding. Simultaneously, Δ^13^C values from the labeled pod were all higher than those from the labeled leaves. Therefore, alfalfa roots were also a C sink, and the assimilated C was primarily transported from the pod during the early stage of seed development. Root regrowth of perennial forage requires an accumulation of nutrients, which likely explains roots as a sink for C in alfalfa. However, with the requirements of roots, competition is likely between roots and seed development for the C contributed by pod photosynthesis; whether the nutrient accumulation by roots is one factor that affects seed yield requires further exploration.

## Conclusions

Based on the results of this experiment, the photosynthetic capacity of alfalfa pods is particularly important during early pod and seed development, and both leaf and pod photosynthesis contribute to meet the carbon requirements of the alfalfa organs of seeds, pods, leaves, stems and roots. The assimilated ^13^C of the pod was used to meet the growth requirements of seeds and pods within the plant. During the development and maturation of the seeds, the requirement for assimilated C in the seed came primarily from the young pod early in development, but in the later stages of development, the leaves became the primary source of C.

## Materials and Methods

### Plant material and pre-treatment

The alfalfa variety Zhongmu No. 2 (*Medicago sativa* L.) was seeded in pots (diameter 50 cm, height 60 cm) in 2012. The pots were set outside, and the seedlings grew in the open environment. Before ^13^C labeling, branches were clipped to have an inflorescence and three leaves underneath. The branch and pod or leaves were wrapped with aluminum foil, and the ^13^C labeling was performed in the dark.

### ^13^C labeling procedures

**Figure 3:**
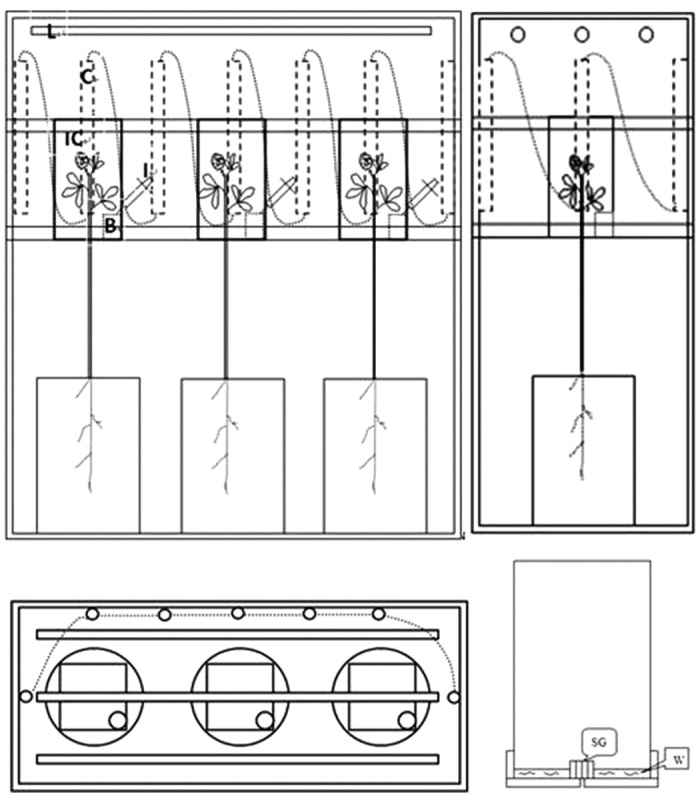
^13^C labeling case of photosynthesis. The material of case is lightproof black cloth or PVC (120 cm × 60 cm × 160 cm), and the inner chamber is high-transparency PVC. L, light (Philips lifemax TLD 30W/54 - 765 Cool DAY LIGHT, made in China) provided a photon flux close to 3 × 1825 lm, and 61 lm/W). C, condensation pipe. IC, inner chamber (11.5 cm × 11.5 cm × 23 cm). SG, sealing gasket (soft rubber). W, water. B, beaker (50 ml). I, injector (20 ml).

Short-term ^13^CO_2_ labeling was used to assess the partitioning of assimilated carbon^16^,^22^,^23^. Labeling of a pod or leaves was performed at the following four stages of pod development after podding: 10, 15, 20 and 25 d. Samples were placed in a self-made controlled environment chamber (Fig. 3), which was a closed system designed for ^13^C labeling. For environmental control, water flow in a spiral condenser was used to maintain the temperature at 25 °C and RH at 70%. The samples were kept in the dark for dark respiration for 30 min, and then a light source was turned on to expose plants for 90 min to ^13^CO_2_-enriched air at a CO_2_ concentration of 1000 μmol mol^−1^ air. This concentration was achieved by supplying a concentration of 640 μmol mol^−1^ CO_2_ to the chamber, which was in addition to the ambient air concentration of CO_2_ of 360 μmol mol^−1^. The concentration was obtained using the following equation: Na_2_^13^CO_3_ (99 atom% ^13^C, 13.95 mg) + H_2_SO_4_ (3 mol l^−1^, Excess) = Na_2_SO_4_ + CO_2_ + H_2_O. The light source was three discharge lamps (Philips Lifemax TLD 30W/54-765 Cool DAY LIGHT, made in China), which had an approximate photon flux of 3 × 1825 lm and 61 lm/W (Figure S3).

### Sampling

**Figure 4:**
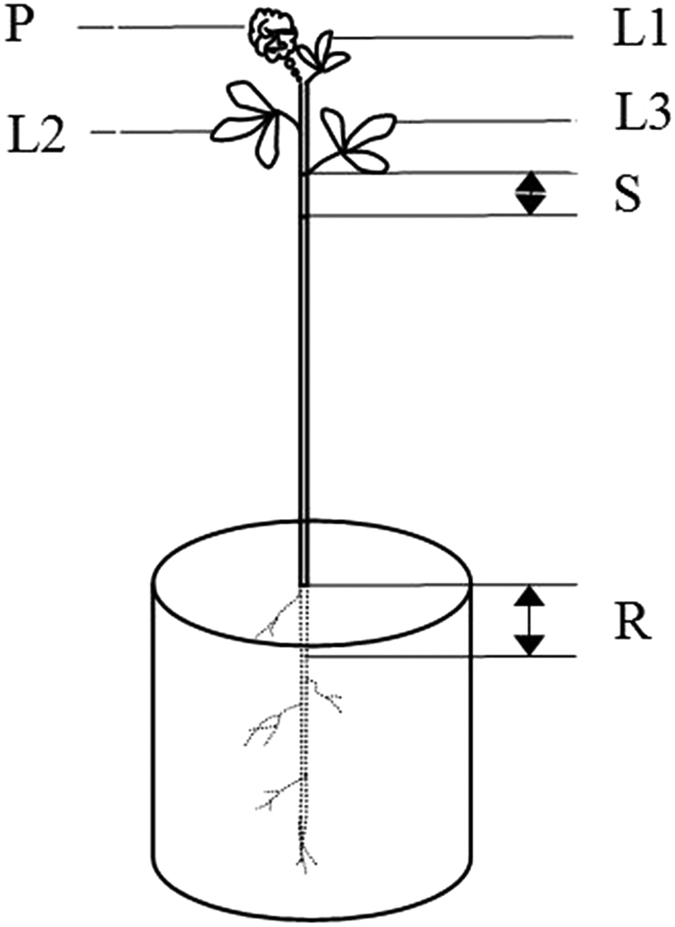
Sampling location of alfalfa after ^13^CO_2_ labeling. P, pod; L1, the first leaf under pod; L2, the second leaf under pod; L3, the third leaf under pod; S, the first internode length stem under the third leaf; R, the 10 cm root under the ground.

At the four stages of pod development, three plants were collected immediately after the labeling period. Additionally, three unlabeled plants were simultaneously harvested to determine baseline ^13^Cabundance. Unlabeled and labeled plants were separated into P, SEED, L, L2, L3, S and R per plant (Fig. 4). These samples were mixed and weighed. The samples were dried rapidly (beginning at 105 °C for 30 min and then increasing to 80 °C for 10 h), weighed and ground to a fine, homogenous powder with a mortar. Powders were stored in the dark within airtight vials until isotopic measurements (Figure S4).

### Isotopic analyses and calculations

The ^13^C/^12^C isotopic ratios of unlabeled and labeled samples were measured with an elemental analyzer (Flash EA 1112, Thermo Electron SPA, USA) coupled to a Thermo Finnigan MAT DELTA^plus^ XP isotopic ratio mass spectrometer (Thermo Finnigan, San Diego, CA, USA) at the Stable Isotope Laboratory for Ecological and Environmental Research, Institute of Botany of the Chinese Academy of Sciences. Isotopic calculations were based on the C isotopic composition of the sample at the end of labeling.

The isotopic signature of C in samples was expressed as δ^13^C (‰). The change of δ^13^C (‰) in samples was expressed as Δ^13^C (‰) = δ_L_^13^C (‰) − δ_N_^13^C (‰), where δ_L_^13^C (‰) and δ_N_^13^C (‰) denote labeled δ^13^C (‰) and unlabeled δ^13^C (‰), respectively.

Distribution of newly assimilated ^13^C atoms within a pod or leaves was expressed as the contribution ratio (CR). Additionally, the CR was expressed as a percentage of the ^13^C in an organ divided by the total ^13^C in the plant. The CR of the pod or leaves to the various organs was assessed by the following equation:


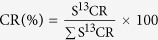
, where S^13^_CR_ represents the Δ^13^C of the sample.

### Statistical analyses

Statistical analyses of the data were conducted using one-way ANOVA and Duncan’s Multiple Range Test (DMRT) at 1% probability to compare the means of different treatments and t-tests at 1% probability to compare the means of samples from the labeled pod and leaves. All analyses were performed with the SPSS 13.0 statistical software package for Windows (SPSS, Inc., Chicago, IL, USA).

## Additional Information

**How to cite this article**: Zhang, W. *et al*. Assessing of the contributions of pod photosynthesis to carbon acquisition of seed in alfalfa (*Medicago sativa*L.). *Sci. Rep.* **7**, 42026; doi: 10.1038/srep42026 (2017).

**Publisher's note:** Springer Nature remains neutral with regard to jurisdictional claims in published maps and institutional affiliations.

## Supplementary Material

Supplementary Information
