# Supplementary material for: Assessing of the contributions of pod photosynthesis to carbon acquisition of seed in alfalfa (Medicago sativa L.)
Source: Sci Rep. 2017 Feb 7;7:42026. doi: 10.1038/srep42026 (PMC5294575; doi:10.1038/srep42026)
Supplement: Supplementary Information [file srep42026-s1.pdf]

Title: Assessing of the contributions of pod photosynthesis to carbon acquisition of seed in alfalfa (*Medicago sativa* L.)

Running title: Carbon acquisition by alfalfa pod

Wenxu Zhang[1,*], Peisheng Mao[2,*], Yuan Li[3], Mingya Wang[2], Fangshan Xia[2] and Hui Wang[2]

[1] Key Laboratory of Eco-Environment-Related Polymer Materials, Ministry of Education of China, Key Laboratory of Gansu Polymer Materials, College of Chemistry and Chemical Engineering, Northwest Normal University, Lanzhou 730070, China

[2] Institute of Grassland Science, China Agricultural University, Beijing 100193, China.

[3] Dryland Farming Institute, Hebei Academy of Agricultural and Forestry Sciences, Hengshui 053000, China

*Corresponding author:

Dr. Wenxu Zhang

Key Laboratory of Eco-Environment-Related Polymer Materials, Ministry of Education of China, Key Laboratory of Gansu Polymer Materials, College of Chemistry and Chemical Engineering, Northwest Normal University

967 Anning East Road, Anning District, Lanzhou 730070, China

Email: zhangwenxu82@163.com

Prof. Peisheng Mao

Institute of Grassland Science, China Agricultural University

2 Yuanming Yuan East Road, Haidian District, Beijing 100193, China.

Email: maops@cau.edu.cn

Fax: +86 10 62739911

Text and display items: abstract 196 words, main text 2846 words, Methods 677 words, 23 references, Figs 1-4

Supplementary information: Appendix figures S1-S4

Type of paper: Articles

Key words: pod, photosynthesis, carbon partitioning, seed, alfalfa.

Online supplementary information: Appendix figures S1-S4

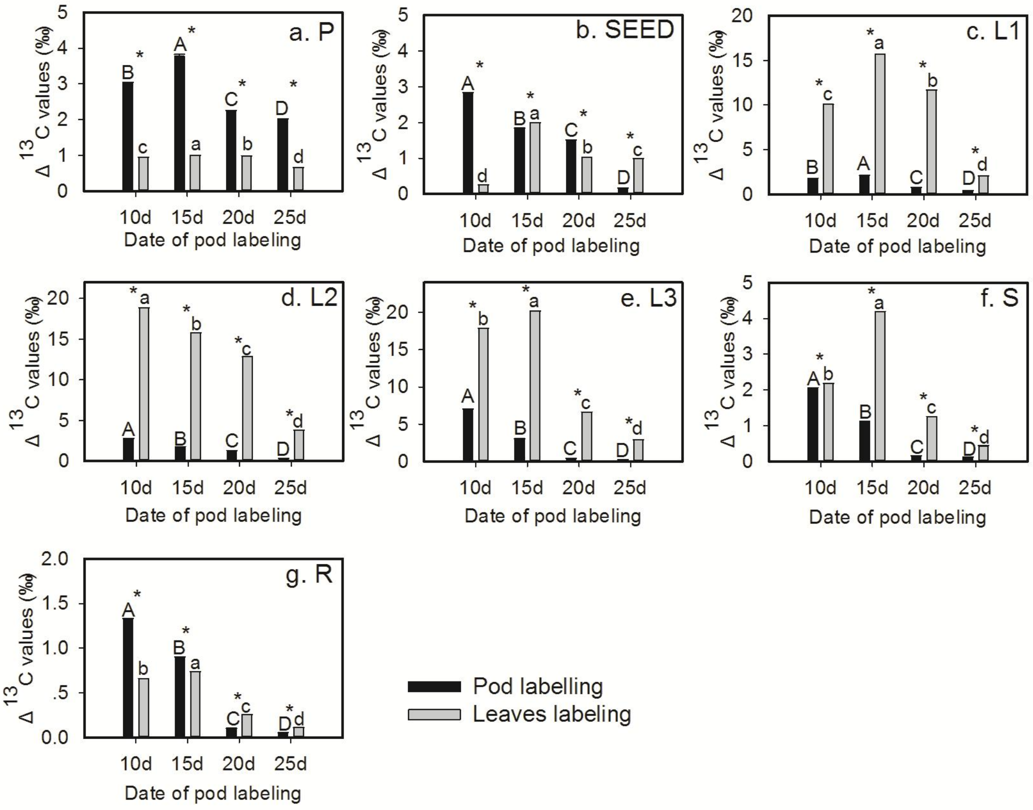

Fig. S1 Changes for$\Delta^{13}C$ values (‰) in the alfalfa organs (P, SEED, L1, L2, L3, S and R) according to the development stage (from day10 to 20 after podding) after pod or leaves labeling. Each value represents the mean±SE (n=3). The different letters indicate significant differences (*P*<0.01) among treatments as determined by the Tukey b-test. The capital and lowcase letter indicates pod and leaves labeling, respectively. * indicate significant differences (*P*<0.01) between pod and leaves labeling of samples as determined by the T test.

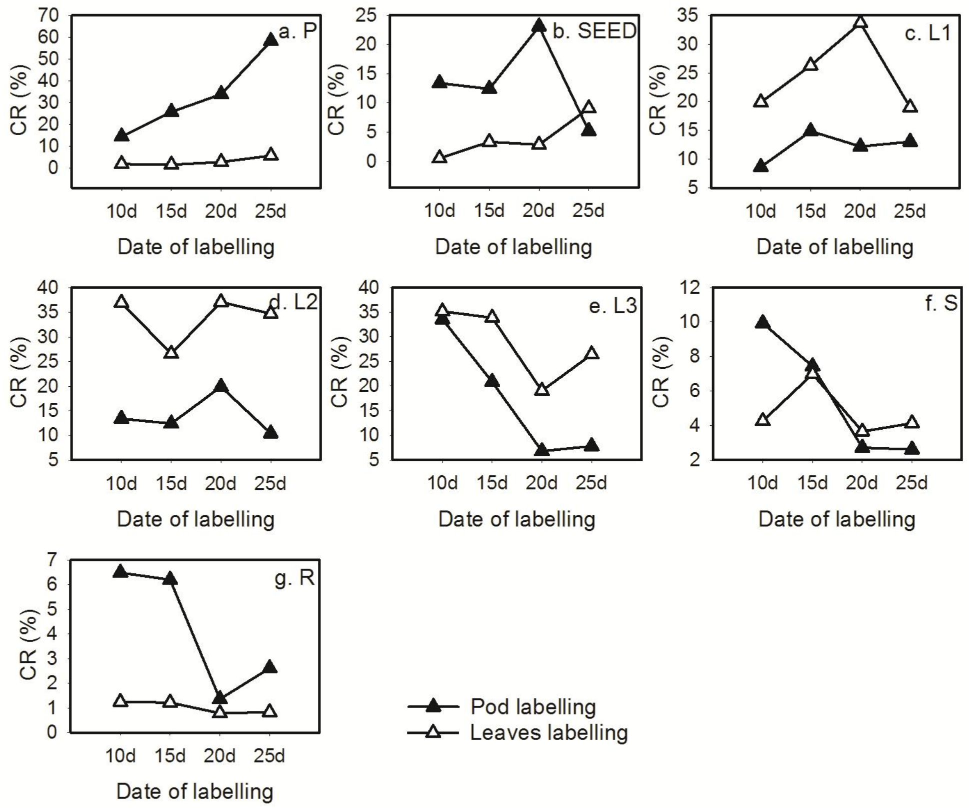

Fig. 2 Changes for the CR of alfalfa organs (P, SEED, L1, L2, L3, S and R) according to the development stage (from day10 to 20 after podding) after pod or leaves labeling.

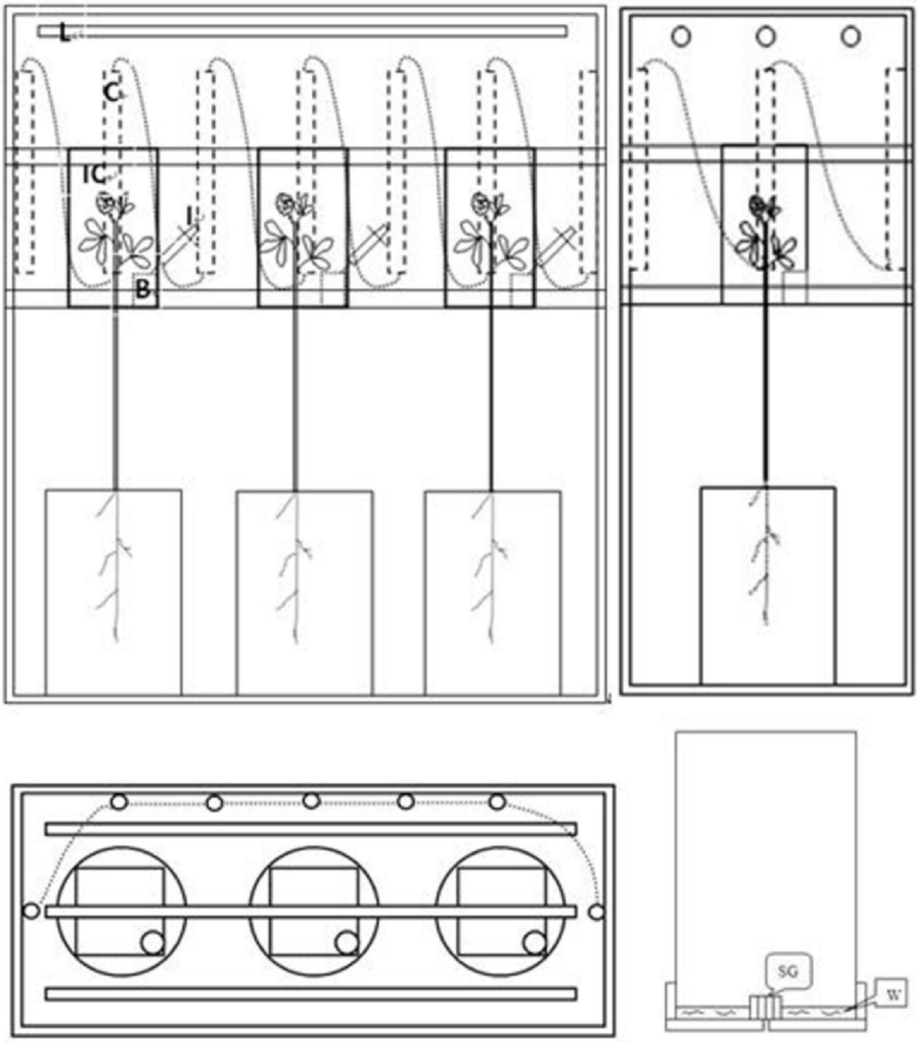

Fig. S3 ^{13}C labeling case of photosynthesis. The material of case is lightproof black cloth or PVC（120cm×60 cm×160 cm）, and the inner chamber is high-transparency PVC. L, light (Philips lifemax TLD 30W / 54 - 765 Cool DAY LIGHT, made in China) provided a photon flux close to 3×1825 lm, and 61 lm / W). C, condensation pipe. IC, inner chamber (11.5 cm×11.5 cm×23 cm). SG, sealing gasket (soft rubber). W, water. B, beaker (50ml). I, injector (20ml).

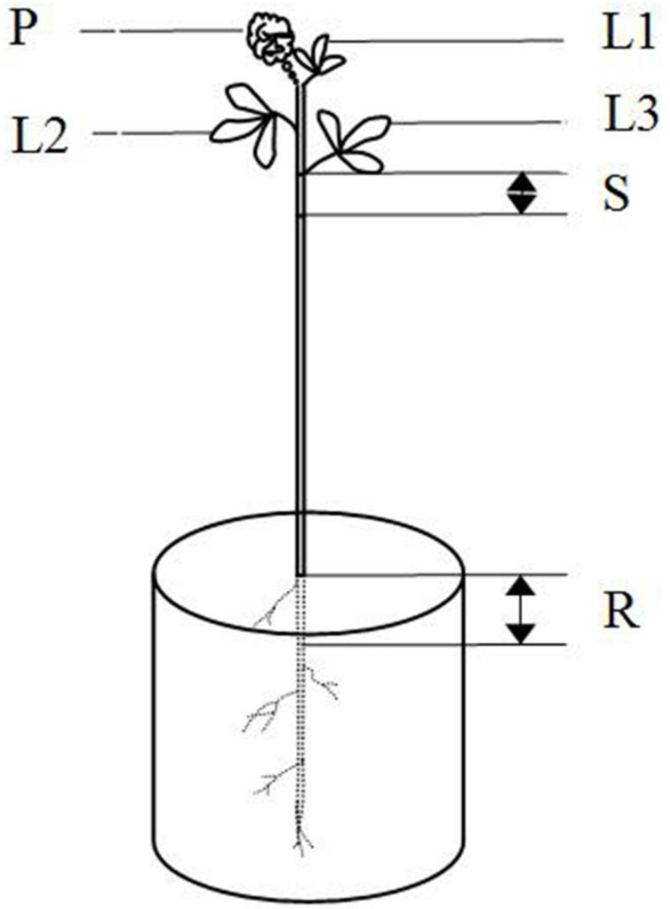

Fig. S4 Sampling location of alfalfa after $^{13}CO_2$ labeling. P, pod; L1, the first leaf under pod; L2, the second leaf under pod; L3, the third leaf under pod; S, the first internode length stem under the third leaf; R, the 10 cm root under the ground.
